# Complete Genome Sequence of a Porcine Reproductive and Respiratory Syndrome Virus Variant with a New Deletion in the 5′ Untranslated Region

**DOI:** 10.1128/genomeA.00090-12

**Published:** 2013-01-24

**Authors:** Kaichuang Shi, Chun’ai Tao, Changhua Lin, Gang Li, Jun Li

**Affiliations:** a Guangxi Center for Animal Disease Control and Prevention, Nanning, China; b Institute of Animal Sciences, Chinese Academy of Agricultural Sciences, Beijing, China

## Abstract

The GX1002 strain is a highly pathogenic porcine reproductive and respiratory syndrome virus (HP-PRRSV) characterized by a continuous 2-nucleotide deletion at positions 119 and 120 in the 5′ untranslated region. This differs from prevalent HP-PRRSVs in China, which have a deletion of only 1 nucleotide at position 119. Here we report the complete genome sequence of the GX1002 strain.

## GENOME ANNOUNCEMENT

Porcine reproductive and respiratory syndrome (PRRS) has caused great economic losses to the swine industry worldwide since it emerged in the late 1980s. The etiologic agent, PRRS virus (PRRSV), which belongs to the family *Arteriviridae*, is an enveloped, single-stranded positive-sense RNA virus. In China, the first North American (NA) type PRRSV strain CH-1a was isolated in 1996 (1). Then the first highly pathogenic PRRSV (HP–PRRSV) variant JXA1, characterized by a discontinuous 30-amino-acid deletion (amino acids [aa] 481 and 533 to 561) in its Nsp2-coding region, was isolated in 2006 (2), and the European (EU) type PRRSV was recently reported (3). Therefore, the epidemics of PRRSVs in China are very complicated, although the HP-PRRSVs (highly pathogenic porcine reproductive and respiratory syndrome viruses) have remained prominent since 2006 (4). Furthermore, PRRSV has shown remarkable genetic variation through mutation or recombination, resulting in the emergence of novel variants (5–8). Here, we report the complete genome sequence of the HP-PRRSV variant GX1002.

The GX1002 strain was isolated from the lung of an infected piglet in a farrow-to-finish unvaccinated farm in Guangxi province, southern China. This farm suffered a severe outbreak of PRRS, with 51.85% (308/594) morbidity and 34.68% (206/594) mortality, in November 2010. To determine its complete genome sequence, fourteen pairs of specific primers were used to amplify 14 overlapped fragments of the GX1002 genome by reverse transcription (RT)-PCR. The PCR products were purified, cloned into a pMD18-T vector (Takara, China), sequenced three times with an ABI3730XL genome sequencer, and assembled into the full-length sequence with SeqMan software (DNAStar Inc.). The terminal sequences were acquired by rapid amplification of cDNA ends using a RACE kit (TaKaRa, China). DNASTAR version 7.0 and Clustal X 2.1 were applied to the genomic analysis. As a result, excluding the poly(A) tail, the complete genome sequence of GX1002 is 15,318 nucleotides (nt) in length, with a 5′ untranslated region (UTR) of 188 nt, followed by a polyprotein precursor coding sequence and a 3′ UTR of 150 nt. GX1002 shares 89.4%, 61.7%, and 99.2% nucleotide identity with the NA prototype VR-2332, the EU prototype Lelystad virus (LV), and the representative HP–PRRSV strain JXA1, respectively. Compared with VR-2332, GX1002 has a discontinuous 30-aa deletion at aa 481 and 533 to 561 of the Nsp2-coding region, indicating that it belongs to HP-PRRSV. Compared with JXA1, GX1002 has multiple genomic variations in different regions, with 110 nt mutations resulting in 56 aa substitutions. Notably, GX1002 has a continuous 2-nucleotide deletion at positions 119 and 120 in the 5′ UTR, which is different from most HP-PRRSV isolates, with only one deletion at position 119 compared with VR-2332 (6, 9). To our knowledge, the HP–PRRSV variant BJPG (GenBank accession no. FJ950746) also has an nt 119 to 120 deletion, suggesting that this deletion in GX1002 is not an isolated case. Since the 5′ UTR of PRRSV plays vital roles in replication, mRNA transcription, and protein translation (10), the additional deletion at nt 120 in the 5′ UTR of GX1002 might affect its infectivity in pigs, which needs further investigation. The genome data of GX1002 will be helpful for understanding the epidemiology, evolution and pathogenesis of PRRSV in pigs.

### Nucleotide sequence accession number.

The complete genome sequence of strain GX1002 is available in GenBank under accession number JQ955658.
